# Social trust predicts sleep disorder at 6 years after the Great East Japan earthquake: data from a prospective cohort study

**DOI:** 10.1186/s40359-020-00436-y

**Published:** 2020-07-01

**Authors:** Yumi Sugawara, Yasutake Tomata, Takuya Sekiguchi, Yutaka Yabe, Yoshihiro Hagiwara, Ichiro Tsuji

**Affiliations:** 1 Division of Epidemiology, Department of Health Informatics and Public Health, Tohoku University School of Public Health, Graduate School of Medicine, 2-1, Seiryo-machi, Aoba-ku, Sendai, Miyagi 980-8575 Japan; 2 Department of Orthopaedic Surgery, Tohoku University School of Medicine, 2-1, Seiryo-machi, Aobaku, Sendai, Miyagi 980-8574 Japan

**Keywords:** Great East Japan earthquake, Social trust, Sleep disorder, Prospective cohort study, Linear mixed model

## Abstract

**Background:**

The physical and psychological health impacts on victims of the Great East Japan Earthquake (GEJE) have lasted for a long time. Some cross-sectional studies have reported a relationship between social networks and/or social support and mental health among victims. Previous studies were cross-sectional observations at one time point after a disaster, it remains unclear whether the lack of social trust soon after the GEJE predicts long-term mental health outcomes among the victims. The objective of the present study was to examine prospectively the association between social trust soon after the GEJE and trends in sleep disorders up to 6 years after the GEJE.

**Methods:**

We conducted a health survey on residents living in two areas affected by the GEJE. We analyzed data from 1293 adults (aged ≥18 years) who had participated in an initial health survey. The participants responded to a self-administrated questionnaire composed of items on health condition, mental health, including sleep disorders (based on the Athens Insomnia Scale [AIS]), and social trust. We classified the participants into two categories (high or low) based on the level of social trust at the first health survey. A linear mixed model was used to estimate trends in AIS scores in relation to social trust at the first health survey.

**Results:**

The AIS scores of participants in the low social trust group were significantly higher than those in the high social trust group throughout the 6 years after the GEJE (*P* < 0.01). After adjusting for some covariates, the AIS score estimate for the participants who had low social trust was 1.30 point higher than those for the participants who had high social trust.

**Conclusion:**

Social trust at 3 to 5 months after the GEJE predicted AIS scores at 6 years after the GEJE among victims. This finding suggests that it may be possible to identify people who have a lower potential for mental resilience from disaster damage over the long term. Further, health interventions for this high-risk group could help promote resilience after a disaster.

## Background

The Great East Japan Earthquake (GEJE) and subsequent giant tsunami that occurred on March 11, 2011 severely damaged the northeast coast of Japan, leaving up to 15,897 people dead and another 2533 missing [1].

The physical and psychological health impacts on victims of the GEJE have lasted for a long time [2–5]. Some studies have suggested that natural disaster victims often experience mental problems such as sleep disorders [6, 7]. Chronic sleep disorder is a well-known risk factor of diseases as cardiovascular disease, diabetes, hypertension, and depression [8–11]. If a better understanding were to be gained of the factors associated with sleep disorders in the early post-disaster period, resilience among disaster victims could be promoted.

Several epidemiologic studies have reported an association between social trust and health outcome [12–16]. Kawachi et al. reported that lower levels of social trust were associated with higher mortality risk [12]. Hyyppa et al. reported that social trust predicted all-cause mortality among adult men and women in Finland [13]. In Japan, one study suggested that women living in communities with higher mistrust had a 1.68 times higher risk of disability onset [14]. Also, two prospective studies reported that social trust might be attenuate hazardous chronic stress or depressed mood [15, 16]. Likewise, some social surveys suggested that social trust was associated with physical or psychological health status [17–19]. For example, Helliwell et al. had reported an association between several trust measures (i.e. general social trust, trust in management, trust in co-workers, trust in neighbours and trust in police) and subjective well-being, based on data from the Gallup World Poll and cycle 17 of the Canadian General Social Survey (GSS17) [18]. Hamamura et al. reported that generalized trust was associated with physical health, happiness and life satisfaction using data from the World Values Survey [19].

Additionally, some cross-sectional studies have reported a relationship between social networks and/or social support and mental health among victims [20–24]. Two cross-sectional studies identified an association between a lack of neighborhood social networks and mental health problems after a disaster [20, 21]. In addition, three cross-sectional studies showed that social support was related to reduced psychological distress among victims [22–24]. For example, Teramoto et al. reported that social companionship from friends in temporary housing complexes was related to fewer psychological problems among displaced earthquake survivors [22]. However, all of these previous studies were cross-sectional observations at one time point after a disaster, and thus, it remains unclear whether the lack of social trust soon after the GEJE predicts long-term mental health outcomes among the victims.

Therefore, the objectives of the present prospective cohort study were to investigate the association between social trust relatively soon after the GEJE and the trends in sleep disorders up to 6 years after the GEJE, and to examine whether social trust predicts long-term sleep disorders in areas affected by the GEJE.

## Methods

### Health survey on victims

We conducted a health survey on residents living in the Ogatsu and Oshika districts of Ishinomaki city, Miyagi Prefecture, which were severely affected by the GEJE. The details of this health survey have been reported elsewhere [25–27]. The health survey on victims consisted of a physical examination and self-reported questionnaires, which were carried out every year since the GEJE. The questionnaires are composed of various items: residence status, medical history, physical health status, smoking habits, drinking habits, dietary habits, sleep status (based on the 8-item Athens Insomnia Scale [AIS]) [28], psychological distress (based on the 6-item Kessler Psychological Distress Scale [K6]) [29], working status, social networks (based on the 6-item Lubben Social Network Scale [LSNS-6]) [30], social trust, norms, and networks.

### Ethical issues

The study protocol was approved by the Institutional Review Board of the Tohoku University Graduate School of Medicine (approval No.: 2011–92, 2017–1-069). Consent to participate in the study was obtained from the participants either face-to-face or via the signed self-administered questionnaires.

### Study participants

**Fig. 1:**
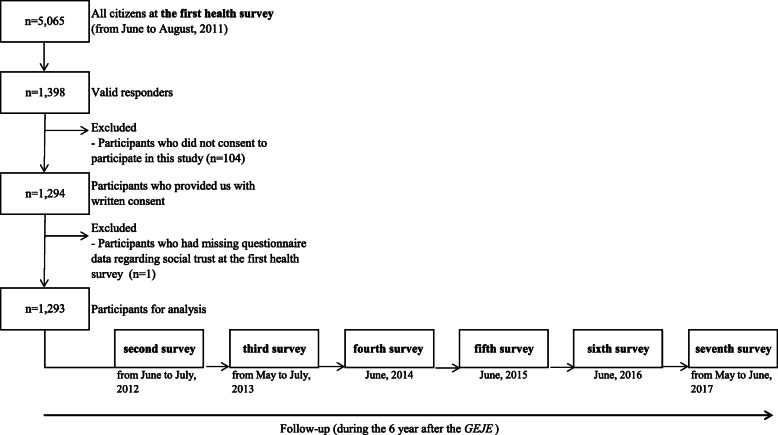
Flow diagram

Of the 5065 residents in this area, 1398 provided valid responses to the initial health survey, which was carried out from June to August 2011. For the present analysis, we excluded residents who did not provide consent to participate (*N* = 104) and who had missing data regarding social trust (N = 1). Finally, 1293 adults were included in the study cohort. Follow-up data were then collected every year after the GEJE (Fig. 1).

### Exposure data

We obtained data on social trust by asking whether “People in my community can be trusted” at the first health survey. The responses were given on a five-point scale (“Strongly agree”, “Somewhat agree”, “Neither agree nor disagree”, “Somewhat disagree”, and “Strongly disagree”). The data were then analyzed with regard to the relation between social trust and sleep disorders, and based on the results, the participants were categorized into two groups: 1) High; those who answered “Strongly agree” or “Somewhat agree”, and 2) Low; those who answered “Neither agree nor disagree”, “Somewhat disagree”, or “Strongly disagree. To assess the reliability of the questionnaire, we calculated the Spearman correlation coefficient (95% confidence interval) between the two surveys (first survey and second survey). The Spearman correlation coefficient for social trust was 0.47 (*p* < 0.01).

### Outcome variables

The outcome variables for sleep disorders were measured using the AIS [28], which is a self-assessment instrument to report any sleep disorders experienced by the participant, provided that they occurred at least three times per week during the last month. The AIS is composed of eight items rated from 0 to 3, with a total score ranging from 0 to 24 [28].

### Other measurements

Residence status was assessed in the questionnaire by selecting one of the following responses: “same as that before the GEJE (no relocation)”, “shelter”, “temporary house”, “rental house”, “family’s house or friend’s house” and “others”. Resident status after the GEJE was categorized into two groups: same housing as that before the GEJE, or relocation after the GEJE.

Economic status was assessed by responses to the following question: “How do you feel about your current house-hold economic status?” The respondents were asked to choose one of the following responses: “very hard”, “hard”, “a little hard” or “normal”. Economic status was categorized into two groups: “very hard” and “hard”, or “a little hard” and “normal”.

Psychological distress was measured using the K6, which is composed of six items rated from 0 to 4, with a total score ranging from 0 to 24 [29].

Social networks were measured using the LSNS-6 [30, 31], which is composed of six items rated from 0 to 5, with a total score ranging from 0 to 30. .

### Statistical analysis

First, we analyzed mean the AIS scores during the 6 years since the GEJE across the social trust groups. The mean AIS scores at every health survey were compared using *t* tests. We then investigated the association between social trust and sleep disorders using a linear mixed model (random effects model). To estimate the effects of sleep status according to social trust at the first health survey, the time from the GEJE (in months) and social trust × time from the GEJE (in months) were entered into the model.

We considered the following variables as possible confounders: sex, age (continuous variable), resident status after the GEJE (same as that before the GEJE, or relocating after the GEJE), and economic status (very hard, hard, a little hard, or normal) (Model 1). These items were chosen as covariates because living environment and economic status may contribute to sleep status. We also added the LSNS-6 score as a social factor because social isolation may affect the association between social trust and sleep status (Model 2) [32, 33]. We further added the K6 score because psychological distress may strongly affect the association between social trust and sleep status (Model 3).

We conducted further stratified analyses according to age group (< 65 years and ≥ 65 years), because age-related differences have been observed in terms of one’s connection with the local community [27].

All *P* values were two-sided, and *P* < 0.05 was considered statistically significant. All statistical analyses were performed using the SAS statistical software package (version 9.4; SAS Institute Inc., Cary, NC, USA).

## Results

Table 1 shows the baseline characteristics of the study participants according to social trust at the first health survey. In the first health survey, 840 (65.0%) of the participants had high social trust, and 453 (35.0%) had low social trust. Participants with lower social trust were more likely to move home after the GEJE and to have lower economic status. Furthermore, they were more likely to be current smokers and to have higher AIS scores (≥6 points) and lower LSNS-6 scores (< 12 points). By contrast, participants with higher social trust were less likely to have sleep disorders and to be socially isolated. However, no significant differences were observed between the social trust groups in terms of medical history or lifestyle factors such as drinking and time spent walking.

**Table 1:** Characteristics of participants according to social trust on the first health survey after the GEJE

	Interpersonal trust^a^				
	High		Low		
	(*n* = 840)		(*n* = 453)		*p*
Sex (%)					
Men	373	(44.4%)	220	(48.6%)	0.20
Women	467	(55.6%)	233	(51.4%)	
Age, years (SD)	64.46	(14.35)	58.82	(13.48)	< 0.01
Resident status (%)					
Same as before the GEJE	409	(48.7%)	185	(40.8%)	< 0.05
Relocation after the GEJE^b^	401	(47.7%)	261	(57.6%)	
Economic status					
very hard or hard	230	(27.4%)	211	(46.6%)	< 0.05
a little hard or normal	607	(72.3%)	241	(53.2%)	
History of stroke (%)	15	(1.8%)	10	(2.2%)	0.59
History of hypertension (%)	329	(39.2%)	167	(36.9%)	0.42
History of myocardial infaction (%)	43	(5.1%)	25	(5.5%)	0.76
History of diabetes mellitus (%)	60	(7.1%)	35	(7.7%)	0.70
Current Smoker (%)	149	(17.7%)	110	(24.3%)	< 0.05
Current Drinker (%)	276	(32.9%)	161	(35.5%)	0.34
Time spent walking (%)					
≤ 0.5 h/day	257	(30.6%)	133	(29.4%)	0.14
0.5–1.0 h/day	301	(35.8%)	144	(31.8%)	
≥ 1 h/day	280	(33.3%)	175	(38.6%)	
AIS score (SD)	4.57	(3.60)	6.44	(3.94)	< 0.01
K6 score (SD)	4.32	(4.08)	6.77	(4.99)	< 0.01
LSNS-6 score (SD)	16.02	(5.66)	13.50	(5.73)	< 0.01

*SD* Standard deviation

^a^ Social trust measured by the responding, “People in my community can be trusted” (High; “Strongly agree” and“Somewhat agree”. Low; “Neither agree nor disagree”, “Somewhat disagree”, and “Strongly disagree”)

^b^ Relocation after the GEJE:shelter, temporary house, rental house, family house or friends house, and others

**Fig. 2:**
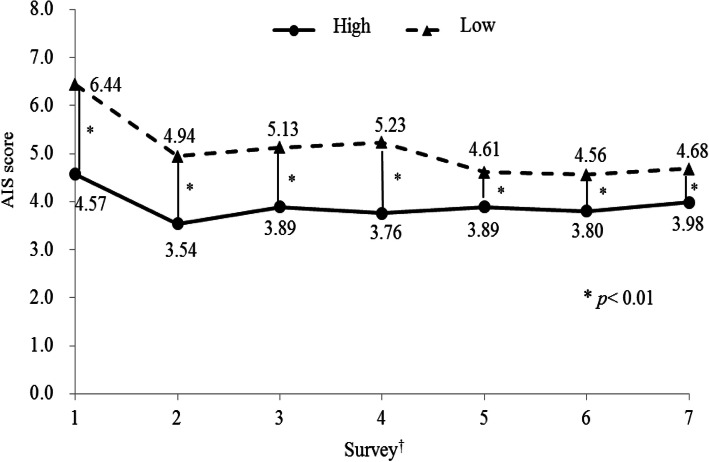
The mean AIS score during follow up fter the Earthquake due to social trust groups at the first survey. †1; First survey - from June to August, 2011. 2; Second survey - from June to July, 2012. 3; Third survey - from May to July, 2013. 4; Fourth survey - June, 2014. 5; Fifth survey - June, 2015. 6; Sixth survey - June, 2016. 7; Seventh survey - from May to June, 2017

Figure 2 shows the mean AIS scores during the 6 years after the GEJE. The mean AIS scores ± standard deviations (SDs) for the high social trust group were 4.57 ± 3.60 in 2011, 3.54 ± 3.23 in 2012, 3.89 ± 3.40 in 2013, 3.76 ± 3.35 in 2014, 3.89 ± 3.39 in 2015, 3.80 ± 3.42 in 2016, and 3.98 ± 3.36 in 2017. The mean AIS scores ± SDs for the low social trust group were 6.44 ± 3.94 in 2011, 4.94 ± 3.87 in 2012, 5.13 ± 3.55 in 2013, 5.23 ± 3.84 in 2014, 4.61 ± 3.39 in 2015, 4.56 ± 3.49 in 2016, and 4.68 ± 3.40 in 2017. The AIS scores of participants in the low social trust group were significantly higher than those in the high social trust group throughout the 6 years after the GEJE (*P* < 0.01).

Table 2 shows the AIS score parameter estimates for the participants in the low social trust group in reference to the high social trust group after the GEJE. After adjusting for covariates, the AIS score parameter estimates for the low social trust group were 1.30 ± 0.18 higher than those for the high social trust group (Model 1). Moreover, the results did not differ when LSNS-6 scores were introduced as a covariate in the linear mixed model (Model 2). To consider the possibility that psychological distress might affect the association between social trust and sleep disorders, we also added the K6 score (continuous variable) to the covariates in the multivariate model (Model 3). However, the results remained essentially unchanged, even after adjusting for psychological distress. The linear mixed model analyses revealed that social trust at the first survey after the GEJE predicted AIS scores during the 6 years after the GEJE. The relationship between social trust after the GEJE and sleep status was independent of social network (LSNS-6) or psychological distress (K6). Additionally, a significant interaction was seen between the social trust group and months from the GEJE. The AIS score among individuals with “high” social trust remained stable throughout the surveys, and that meanwhile among those with “low” interpersonal trust, the AIS score was higher level in the soon after the GEJE and then gradually decreased throughout the surveys (Fig. 2).

**Table 2:** Parameter estimates for AIS score comparing social trust among the victims after the GEJE

		Model1^a^				Model2^b^				Model3^c^			
		Coefficient	SE	t	*p*	Coefficient	SE	t	*p*	Coefficient	SE	t	*p*
Intercept		2.086	0.446	4.68	<.0001	2.184	0.493	4.43	<.0001	1.353	0.387	3.50	<.001
Time from the GEJE (month)		0.023	0.008	3.09	0.002	0.028	0.008	3.35	0.001	0.033	0.007	4.88	<.0001
Social trust	Low	1.301	0.180	7.21	<.0001	1.285	0.184	6.98	<.0001	0.679	0.150	4.54	<.0001
	High	Reference				Reference				Reference			
Variances													
Age		0.009	0.006	1.42	0.157	0.009	0.006	1.43	0.153	0.010	0.005	2.08	0.037
Sex	Female	1.098	0.167	6.57	<.0001	1.099	0.167	6.57	<.0001	0.821	0.137	5.98	<.0001
	Male	Reference				Reference				Reference			
Resident status	Relocation after the GEJE	0.840	0.170	4.95	<.0001	0.846	0.170	4.97	<.0001	0.642	0.139	4.62	<.0001
	Same as before the GEJE	Reference				Reference				Reference			
Economic status	very hard/ hard	1.821	0.182	10.01	<.0001	1.821	0.182	6.98	<.0001	1.105	0.151	7.33	<.0001
	a little hard/normal	Reference				Reference				Reference			
LSNS-6						−0.007	0.015	−0.46	0.647	−0.017	0.009	−1.94	0.053
K6										0.332	0.011	30.21	<.0001
Social trust×time from the GEJE	Low	−0.012	0.003	−4.19	<.0001	−0.013	0.003	−4.37	0.008	−0.007	0.003	−2.84	0.005
	High	Reference				Reference				Reference			

^a^ Model1; adjust for sex, age (continuous variable), time from the GEJE (months), resident status (same as before the GEJE, relocation after the GEJE), economic status (very hard or hard, a little hard or normal)

^b^ Model2; model1added to LSNS-6 score (continuous variable)

^c^ Model3; model2 added to K6 score (continuous variable)

Table 3 shows a comparison of AIS score parameter estimates between the social trust groups divided by age (< 65 years and ≥ 65 years) among the victims of the GEJE. The AIS score parameter estimates were similar for both younger and older participants (< 65 years: 1.39 ± 0.24; ≥65 years: 1.20 ± 0.28). No age differences were observed in the interaction between social trust and months from the GEJE.

**Table 3:** Parameter estimates for social trust accoding to age (< 65 years, ≥65 years) among the victims after the GEJE^a^

		< 65 years								≥65 years							
		(*n* = 651)								(*n* = 642)							
		Model2^a^				Model3^b^				Model2^a^				Model3^b^			
		Coefficient	SE	t	*p*	Coefficient	SE	t	*p*	Coefficient	SE	t	*p*	Coefficient	SE	t	*p*
Intercept		2.434	0.411	5.92	<.0001	1.963	0.287	6.83	<.0001	3.068	0.403	7.62	<.0001	2.059	0.281	7.33	<.0001
Time from the GEJE (month)		0.012	0.007	1.70	0.089	0.017	0.006	2.96	0.003	0.011	0.007	1.66	0.098	0.016	0.006	2.70	0.007
Social trust	Low	1.390	0.244	5.69	<.0001	0.627	0.194	3.24	0.001	1.196	0.281	4.26	<.0001	0.706	0.234	3.02	0.003
	High	Reference				Reference				Reference				Reference			
Variances																	
Sex	Female	0.753	0.230	3.27	0.001	0.458	0.185	2.48	0.013	1.429	0.242	5.91	0.001	1.140	0.203	5.62	<.0001
	Male	Reference				Reference				Reference				Reference			
Resident status	Relocation after the GEJE	1.012	0.235	4.31	0.009	0.831	0.19	4.43	<.0001	0.611	0.248	2.46	0.014	0.427	0.208	2.06	0.040
	Same as before the GEJE	Reference				Reference				Reference				Reference			
Subjective economic status	very hard/ hard	1.662	0.238	6.99	<.0001	0.848	0.193	4.41	<.0001	1.973	0.280	7.04	<.0001	1.333	0.237	5.64	<.0001
	a little hard/normal	Reference				Reference				Reference				Reference			
LSNS6 score (the first survey)		0.020	0.021	0.94	0.347	−0.018	0.012	−1.48	0.138	−0.031	−0.031	−1.47	0.142	−0.014	0.012	−1.18	0.239
K6						0.373	0.016	23.37	<.0001					0.299	0.015	19.79	<.0001
Social trust×time from the GEJE	Low	−0.009	0.004	−2.15	0.031	−0.001	0.003	−0.23	0.817	−0.018	0.005	−3.96	0.034	−0.013	0.004	−3.57	<.001
	High	Reference				Reference				Reference				Reference			

^a^Model2; adjust for sex, time from the GEJE (months), resident status (same as before the GEJE, relocation after the GEJE), economic status (very hard or hard, a little hard or normal), LSNS-6 score (continuous variable)

^b^Model3; model2 added to K6 score (continuous variable)

## Discussion

The present study clarified that social trust 3 to 5 months after the GEJE predicted AIS scores throughout the 6 years after the GEJE. Low social trust was more strongly associated with the AIS score parameter estimates than high social trust, even after adjusting for some covariates. Additionally, when we conducted stratified analyses according to age at the first survey (< 65 years or ≥ 65 years), the results did not change.

A number of previous studies have reported a relation between SC, such as social networks or social support, and mental health among disaster victims in Japan [18–22]. However, these previous studies were cross-sectional observations conducted at one time point after the disaster; to our knowledge, no prospective study has examined the association between social trust and sleep status among victims of the GEJE. In the present study, we demonstrated that a lack of social trust relatively soon after the GEJE predicted sleep disorders about 6 years later. This finding suggests that it may be possible to identify people who have a lower potential for mental resilience over the long term and health intervene is required for people with low social trust in the early post-disaster period.

Kawachi et al. reported that SC is a potentially relevant force in disasters, and described how its mechanisms may indicate the beneficial effects of social connections, which become apparent in the presence of a major stressor [34]. Nakagawa and Shaw examined the role of SC in earthquake rehabilitation and reconstruction programs in two cases—Kobe, Japan and Gujarat, India—and suggested that SC, which is defined as a function of trust, social norms, social participation, and social networks, can play an important role in recovery [35]. The association observed between social trust and mental health among victims in the present study would be attributable to this mechanism.

Moreover, social trust might influence stress coping among survivors who were severely affected by the GEJE. A study from Japan reported that social trust might attenuate hazardous chronic stress by promoting social participations and support [15]. A study from China also reported that social trust was negatively associated with depressed mood, and it would therefore help people build new relationships [16].

We found a significant interaction effect between the social trust group and time from the GEJE. This interaction might be attributable to differences in support for the study subjects during follow-up. In our study, participants with lower social trust were more likely to have lower economic status (Table 1), and these participants might have received more social support or services than those with higher social trust.

Our study had several strengths. First, the present study is a prospective design and used a linear mixed model. The linear mixed model is a well-known a tool for analysis of repeated data, including missing data, by performing the fitting of the model for each subject. We estimated the final time-dependent changes in the sleep status of the participants at 6 years after the GEJE using a linear mixed model by comparing the low and high social trust groups from the first survey. In addition, we followed the victims throughout the 6 years after the GEJE. Second, we used the AIS, which has high validity, to evaluate sleep conditions [28]. The AIS is an effective tool for assessing individual sleep conditions or quality, even when victims had no time to respond after the GEJE.

This study also had some limitations. First, the response rate for the first health survey was low (27.6%); therefore, the respondents might have experienced less severe damage than the non-respondents. Additionally, the present results might have underestimated the association between social trust relatively soon after the GEJE and the trends in sleep disorders up to 6 years after the GEJE. Second, we had no data on the history of mental illness, such as depression, before the GEJE, which could also be associated with the risk of sleep disorders.

## Conclusion

In conclusion, to our knowledge, this is the first study to reveal that social trust was associated with long-term sleep disorders in victims of the GEJE for as long as 6 years. This finding suggests that it may be possible to identify people who have a lower potential for mental resilience from disaster damage over the long term. Further, health interventions for this high-risk group could help promote resilience after a disaster.

## Data Availability

All data generated or analysed during this study are not publicly available, but are available from the corresponding author on reasonable request.

## Abbreviations

GEJE: Great East Japan Earthquake
SC: Social capital
HR: Hazard ratio
CI: Confidence interval
AIS: Athens Insomnia Scale
K6: Kessler 6-item Psychological Distress Scale
LSNS-6: Lubben Social Network Scale
SD: Standard deviation
